# Deletion of sphingosine kinase 1 inhibits liver tumorigenesis in diethylnitrosamine-treated mice

**DOI:** 10.18632/oncotarget.24583

**Published:** 2018-02-26

**Authors:** Jinbiao Chen, Yanfei Qi, Yang Zhao, Dominik Kaczorowski, Timothy A. Couttas, Paul R. Coleman, Anthony S. Don, Patrick Bertolino, Jennifer R. Gamble, Mathew A. Vadas, Pu Xia, Geoffrey W. McCaughan

**Affiliations:** ^1^ Liver Injury and Cancer, Camperdown, NSW 2050, Australia; ^2^ Vascular Biology, Camperdown, NSW 2050, Australia; ^3^ ACRF Centenary Cancer Research, Camperdown, NSW 2050, Australia; ^4^ Liver Immunology in Centenary Institute, Camperdown, NSW 2050, Australia; ^5^ Department of Endocrinology and Metabolism, Zhongshan Hospital, Fudan University, Shanghai 200032, China; ^6^ A.W. Morrow Gastroenterology and Liver Center, Australian Liver Transplant Unit, Royal Prince Alfred Hospital, Camperdown, NSW 2050, Australia; ^7^ Sydney Medical School, University of Sydney, Camperdown, NSW 2050, Australia

**Keywords:** mouse, liver cancer, Sphk1, sphingosine, myc

## Abstract

Primary liver cancer is the 3rd leading cause of cancer deaths worldwide with very few effective treatments. Sphingosine kinase 1 (SphK1), a key regulator of sphingolipid metabolites, is over-expressed in human hepatocellular carcinoma (HCC) and our previous studies have shown that SphK1 is important in liver injury. We aimed to explore the role of SphK1 specifically in liver tumorigenesis using the SphK1 knockout (*SphK1*^−/−^) mouse. SphK1 deletion significantly reduced the number and the size of DEN-induced liver cancers in mice. Mechanistically, fewer proliferating but more apoptotic and senescent cells were detected in SphK1 deficient tumors compared to WT tumors. There was an increase in sphingosine rather than a decrease in sphingosine 1-phosphate (S1P) in SphK1 deficient tumors. Furthermore, the STAT3-S1PR pathway that has been reported previously to mediate the effect of SphK1 on colorectal cancers was not altered by SphK1 deletion in liver cancer. Instead, c-Myc protein expression was down-regulated by SphK1 deletion. In conclusion, this is the first *in vivo* evidence that SphK1 contributes to hepatocarcinogenesis. However, the downstream signaling pathways impacting on the development of HCC via SphK1 are organ specific providing further evidence that simply transferring known oncogenic molecular pathway targeting into HCC is not always valid.

## INTRODUCTION

Hepatocellular carcinoma (HCC), which accounts for nearly 80% of all primary liver cancers, is the 3rd leading cause of cancer deaths worldwide with a steeply rising incidence [1, 2]. HCC tumorigenesis is closely associated with liver chronic injury although detailed mechanisms are yet to be fully explored [3, 4]. The lack of understanding of HCC tumorigenesis hinders the development of effective prevention approaches and better therapies for HCC. Sorafenib, an orally active multi-kinase inhibitor, is the only agent showing survival benefits in patients with advanced HCC [1]. New therapeutic targets for HCC are desperately needed.

Sphingosine kinase (SphK), including SphK1 and SphK2, are key regulators of sphingolipid metabolites [5]. There has been an accumulating body of evidence that SphK1 is critically involved in cancer development since we first reported the oncogenic potential of SphK1 [6–8]. Overexpression or knockout of SphK1 has been shown to enhance or inhibit tumorigenesis, respectively, as exampled in a murine model of colorectal cancer [6, 9–12]. SphK catalyzes phosphorylation of sphingosine to sphingosine-1-phosphate (S1P) that has diverse signalling properties by acting through five S1P receptors (S1PR) or other intracellular targets [5, 7, 13]. However, recent studies show that highly potent and selective SPHK1 inhibitors do not affect cancer cell proliferation or survival thus raising the possibility that regulation through sphingosine itself might play a more important role in oncogenic transformation and tumor growth [11, 14, 15].

Several pieces of information lead to us undertaking this study. Firstly, hepatic steatosis is a risk factor for HCC and we, with others, have shown that deletion of SphK1 ameliorates hepatic steatosis [16, 17]. In addition, SphK1 is highly expressed in HCC [18], suggesting it may be important in liver tumorigenesis. In addition, the molecular basis of HCC development and progression is very heterogeneous. Phase 2/3 clinical human trials derived from the simple transfer of targets from other cancers to HCC development has largely failed over the past decade. Thus, given the known role of Sphk1 in other cancers but not in HCC, we proposed that this pathway needed to be specifically examined in HCC. We aimed to evaluate the role of SphK1 in the development of primary liver cancer by using a mouse model of diethylnitrosamine (DEN)-induced liver cancer in the SphK1 knockout (*SphK1*^*−/−*^) mouse [19, 20]. We show that DEN-induced liver cancers in *SphK1*^*−/−*^ mice were significantly less and smaller than those in wild type (WT) mice. Furthermore, the mechanism underlying this inhibition was different to that reported in colon carcinogenesis and is associated with an increase in sphingosine rather a decrease in S1P. This was then associated with an increase in senescence and apoptosis and a decrease in Myc and cellular proliferation.

## RESULTS

### Expression of SphK1, not SphK2, is up-regulated in liver cancers

**Figure 1:**
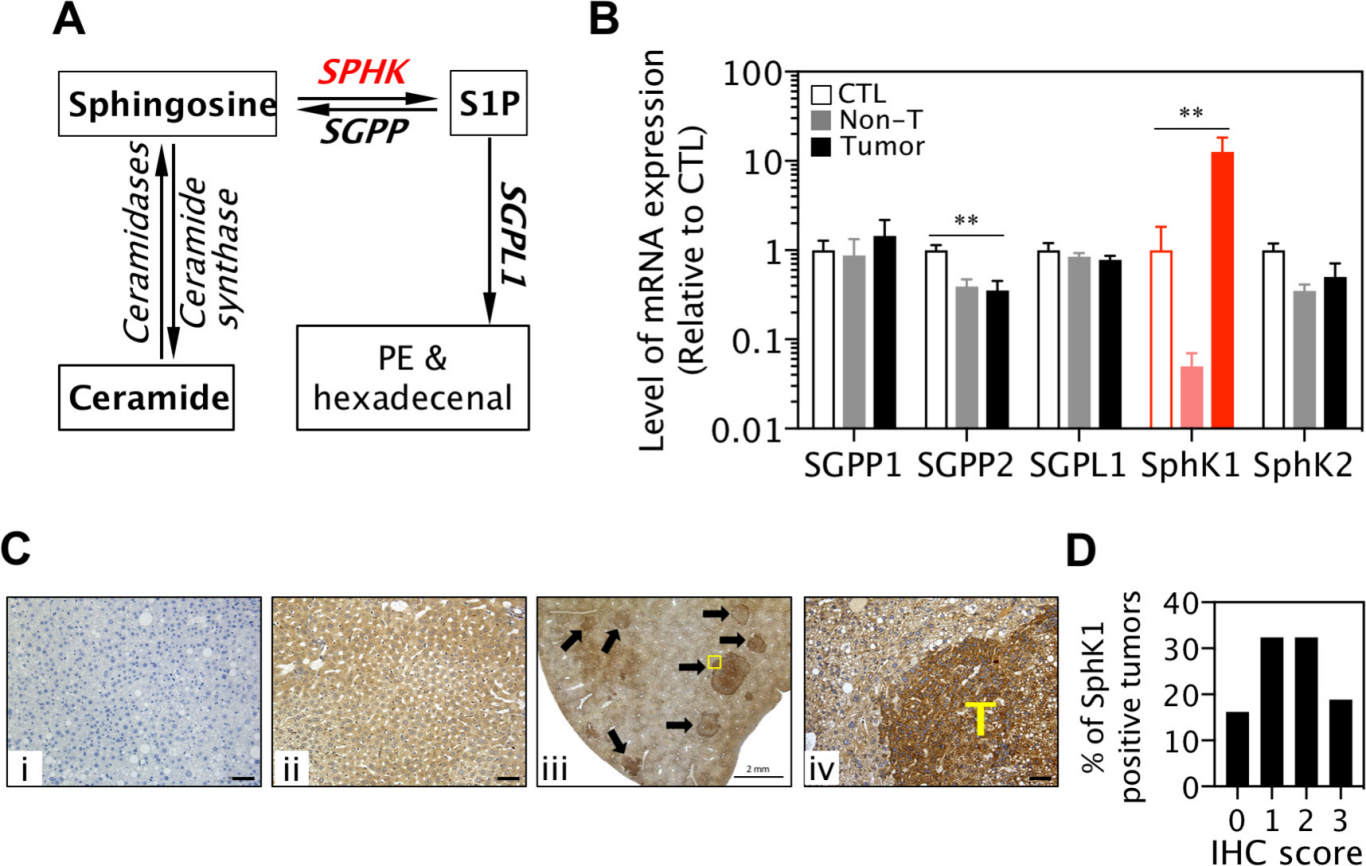
SphK1 expression is increased in liver cancers (**A**) Schematic diagram of ceramide, sphingosine and S1P metabolism. PE: phosphoethanolamine. (**B**) Levels of SGPP, SGPL1 and SphK mRNA expression in livers without DEN treatment (CTL, 8 WT and *SphK1*^*−/−*^), DEN-induced non-tumor livers (non-T) and liver tumors (8 WT and 5 *SphK1*^−*/*−^). ^**^p < 0.01, one-way ANOVA. (**C** and **D**) Expression of SphK1 detected with immunohistochemistry (IHC) in mouse liver cancers: (i) Negative control (the rabbit antibody against SphK1 was omitted, but normal rabbit IgG was applied), (ii) normal mouse liver, (iii & iv) mouse liver cancers (*n* = 11), T: tumor area. Scale bars in i, ii and iv: 50 μm. (d) Score of SphK1 IHC staining (37 tumours).

Among several key enzymes regulating the levels of ceramide, sphingosine and S1P (Figure 1A), SphK1 but not SphK2 mRNA expression was significantly increased in DEN-induced liver cancer tissues compared with liver tissues without DEN treatment. Further, sphingosine-1-phosphatase 2 (Sgpp2), which catalyses dephosphorylation of S1P to sphingosine, was significantly reduced (Figure 1B). Other enzymes such as Sgpp1 and sphingosine-1-phosphate lyase (Sgpl1) were unchanged (Figure 1B). In agreement with published human patient data [18], levels of SphK1 protein expression were significantly higher in liver tumor cells than in surrounding normal hepatocytes (Figure 1C and 1D). These results suggest SphK1 may play a role in the development of liver cancer.

### Deletion of SphK1 reduces DEN-induced liver tumor initiation and progression

**Figure 2:**
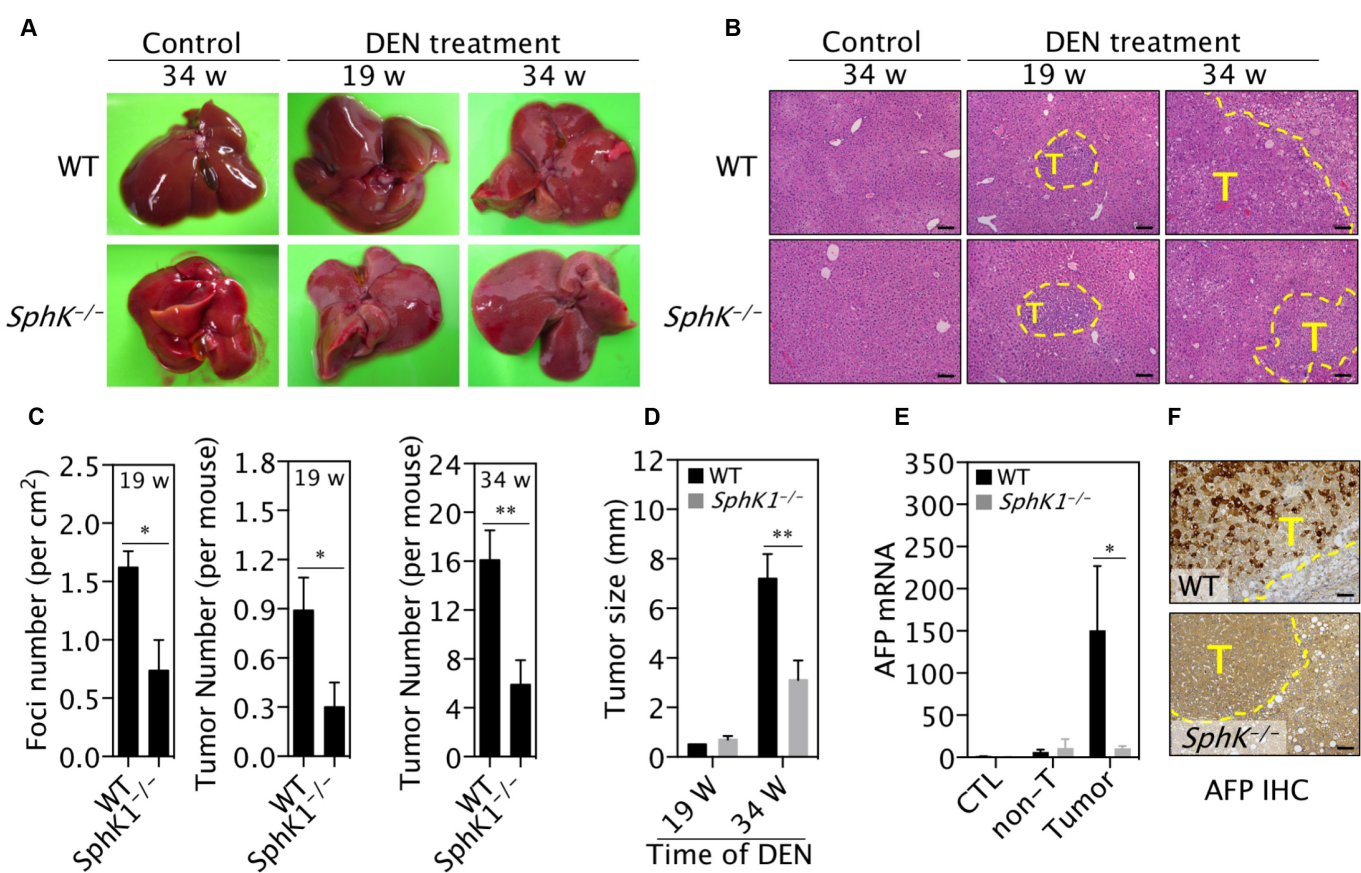
SphK1 deletion inhibits DEN-induced liver tumorigenesis DEN (25 mg/Kg body weight) was given once to WT and *SphK1*^*−/−*^ mice at postnatal day 14. Livers were harvested at 19 and 34 weeks (w) post-DEN injection. Control (CTL) livers were harvested from mice without DEN treatment. (**A**) Representative pictures of livers. Liver tumors are displayed as pale yellowish-white nodules on the surface of livers. (**B**) Representative pictures of liver sections with H&E staining. Tumors are with dashed lines. T: tumor. Scale bars: 100μm. (**C**) The number of liver tumors per mouse (or foci, which was counted under microscope and expressed as per mm^2^ of sections). (**D**) The maximum size of liver tumors. (**E** and **F**) The expression of AFP was determined with (E) qPCR for mRNA and (F) immunohistochemistry for protein. Non-T: non-tumor. Scale bars: 50 μm. Data is represented as mean ± SEM. There were 9 & 10 mice at 19 weeks and 11 & 9 mice at 34 weeks for WT and *SphK1*^*−/−*^ mice, respectively, ^*^p < 0.05, ^**^p < 0.01 (one-way ANOVA).

To further explore the role of SphK1 in liver tumorigenesis, we utilized *SphK1*-knockout strategy in the DEN-induced HCC model [19]. When analysed at both 19 and 34 weeks post-DEN injection the number of DEN-induced macroscopic liver cancers in the *SphK1*^*−/−*^ mice was significantly reduced by 60% compared to the numbers in the WT mice (Figure 2A and middle & right panels in 2C). Microscopic analysis of the liver cancers at 19 weeks post-DEN injection using H&E stained sections (Figure 2B and left panel in 2C) confirmed the significant reduction in the *SphK1*^*−/−*^ mice compared to WT mice. Intriguingly, the proportion of reduction caused by SphK1 deficiency was not changed during the course of experiments, suggesting that less tumors in *SphK1*^*−/−*^ mice is likely attributable to the inhibition of tumor initiation. In addition to a reduction in the number of tumors, the maximum size of tumor was decreased by 60% in *SphK1*^*−/−*^ mice compared to WT at 34 weeks post-DEN injection (Figure 2D). The reduction in tumor load was confirmed by the measurements of mRNA levels of alpha-fetoprotein (AFP), a HCC biomarker, which was significantly reduced in *SphK1*^*−/−*^ compared to WT mice (Figure 2E). Further, less AFP positive cells were found in *SphK1*^*−/−*^ tumors compared to WT tumors (Figure 2F). These results demonstrated that SphK1 deficiency inhibits liver tumor progression in DEN-treated mice.

### Deletion of SphK1 inhibits liver tumor growth

**Figure 3:**
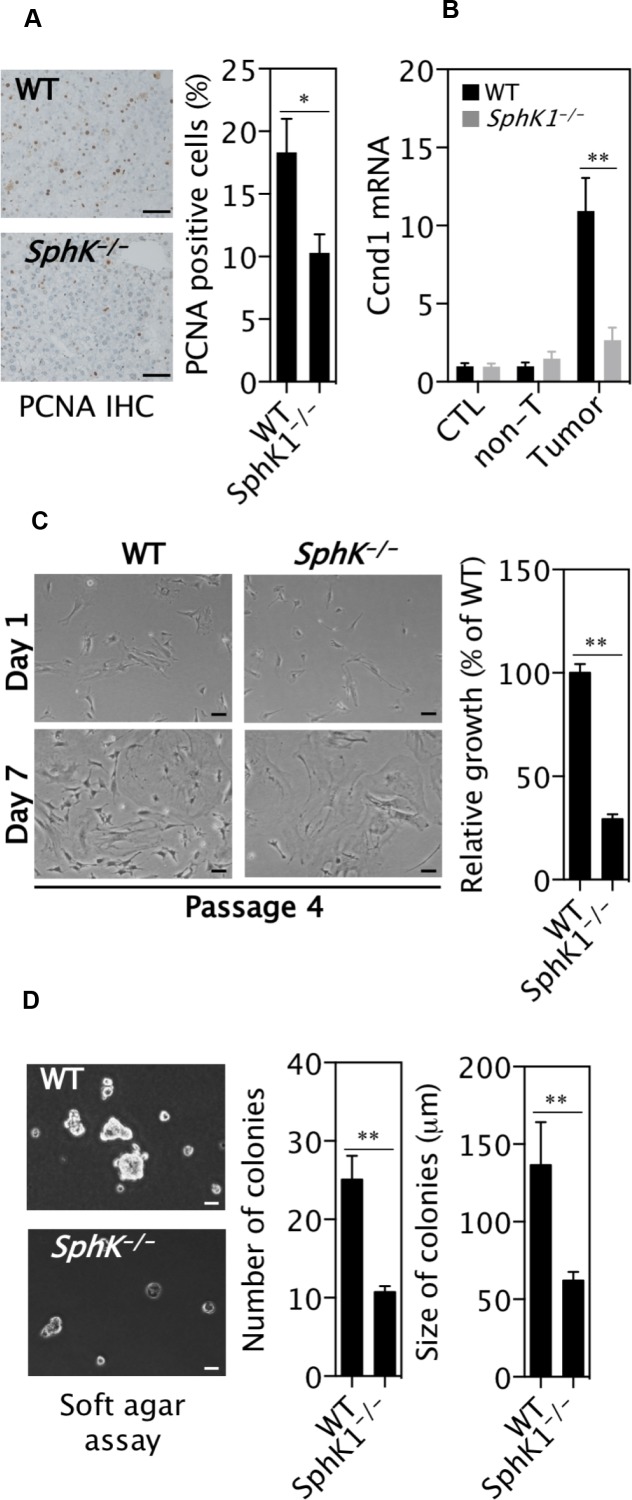
SphK1 deletion inhibits the proliferation of liver cancer cells DEN was given once to WT and *SphK1*^*−/−*^ mice at postnatal day 14. Livers were harvested at 34 weeks post-DEN injection. (**A**) PCNA positive cells in tumors (9 WT and 5 *SphK1*^*−/−*^) detected with immunohistochemical staining. (**B**) Cyclin D1 mRNA expression by RT-qPCR in normal livers (CTL, 8 WT and *SphK1*^*−/−*^), DEN-treated non-tumor livers (non-T) and tumors (8 WT and 5 *SphK1*^*−/−*^). (**C**) The relative growth of primary tumour cells *in vitro* (*n* = 6). (**D**) Soft agar colony formation assay. There were 7 biological repeats with 4 primary cell lines each genotype (One line was tested once and another three lines were tested twice at different passage number). Scale bars: 50 μm. Data represented as mean ± SEM, ^*^p < 0.05, ^**^p < 0.01 (one-way ANOVA).

There were less proliferative cells defined by PCNA protein expression (Figure 3A) and cyclin D1 mRNA expression (Figure 3B) in *SphK1*^*−/−*^ tumors compared to WT tumors. Primary *SphK1*^*−/−*^ tumor cells showed much slower growth than primary WT tumor cells (Figure 3C). Furthermore, long-term survival assays show that *SphK1*^*−/−*^ tumor cells formed fewer and smaller colonies in soft agar than WT tumor cells (Figure 3D), indicating that SphK1^−/−^ tumor cells have less growth potential than WT tumor cells.

### SphK1 deletion promotes cell death and senescence within the tumors

**Figure 4:**
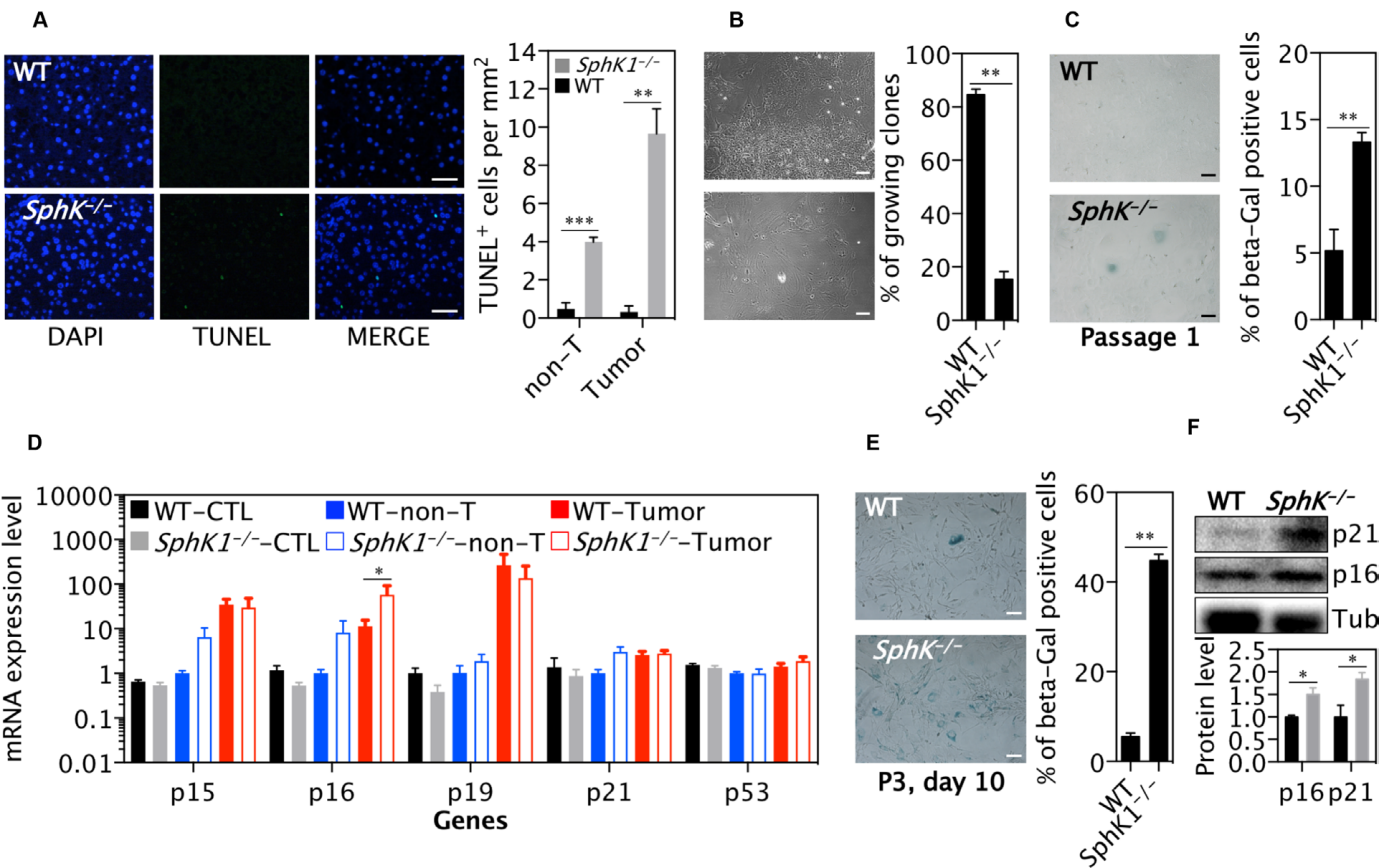
SphK1 deletion increases cellular senescence in primary tumor cells and MEF cells (**A**) TUNEL positive cells in non-tumor (non-T) and tumors in livers (*n* = 8) that were harvested at 34 weeks post-DEN injection. (**B**) The percentage of survival clones of primary tumor cells. The number of primary tumor cell clones was counted at day 3 and 14 after primary tumor cells (passage 0) were seeded. There were 9 and 8 replicates from 3 WT and *SphK1*^*−/−*^ tumors, respectively. (**C**) SA β-Gal staining was performed on primary cancer cells (passage 1). Cells staining positively for SA β-Gal were counted and graphed (*n* = 4). (**D**) Quantitative PCR analysis of mRNA expression of genes in normal livers (CTL, 8 WT and *SphK1*^*−/−*^), non-tumor tissues (non-T) or liver tumors (8 WT and 5 *SphK1*^*−/−*^) harvested at 34 weeks post-DEN injection. (**E**) SA β-Gal staining on WT and *SphK1*^*−/−*^ MEF cells and its quantification (*n* = 4). (**F**) Representative images (top) and quantification (bottom panel) of Western blotting detecting protein expression of genes in MEF (4 WT and 4 *SphK1*^*−/−*^). Data is shown as mean ± SEM, ^*^p < 0.05, ^**^p < 0.01 (one-way ANOVA). Scale bars: 50 μm in A, B and C; 100 μm in A.

In line with the well-established anti-apoptotic effect of SphK1, there were significantly more apoptotic cells detected by TUNEL staining (Figure 4A) in *SphK1*^*−/−*^ tumors than WT tumors. In order to elucidate the mechanisms responsible for the effects of SphK1 deficiency on liver tumor cell death, primary mouse liver tumor cells were isolated and cultured from WT and *SphK1*^*−/−*^ tumors. Remarkably, although primary liver tumor cells from both WT and *SphK1*^*−/−*^ underwent cell death, there was significantly more death in the *SphK1*^*−/−*^ tumor cells as fewer colonies of cells were formed from *SphK1*^*−/−*^ tumor cells than WT tumor cells (Figure 4B). In addition to the enhanced apoptosis, large flattened cells were clearly evident in the cells from the *SphK1*^*−/−*^ tumors, suggesting senescence. This was confirmed by staining with the senescence marker, senescence associated β-galactosidase (SA-β-gal) (Figure 4C). Further, expression of p16 mRNA, a marker of senescent cells, was increased in *SphK1*^*−/−*^ tumor tissues compared to WT tumors (Figure 4D). To verify the effect of SphK1 deletion on activation of the senescence pathway, mouse embryonic fibroblasts (MEFs) were isolated from *SphK1*^*−/−*^ and WT embryos at the same age and cultured using a standard 3T3 protocol [21, 22]. Fewer *SphK1*^*−/−*^ MEFs bypassed the senescence crisis after passage 3 (Figure 4E) and indeed a nearly 5-fold increase in the number of senescent cells was evidenced in *SphK1*^*−/−*^ MEF compared with WT cells (Figure 4E). The expression of p16 and p21 protein was also higher in *SphK1*^*−/−*^ MEF than WT MEF (Figure 4F). These results indicated that senescence was promoted by SphK1 deletion, which together with apoptosis, contribute to the inhibition of liver tumorigenesis.

### Deletion of SphK1 increases the level of sphingosine in tumors

**Figure 5:**
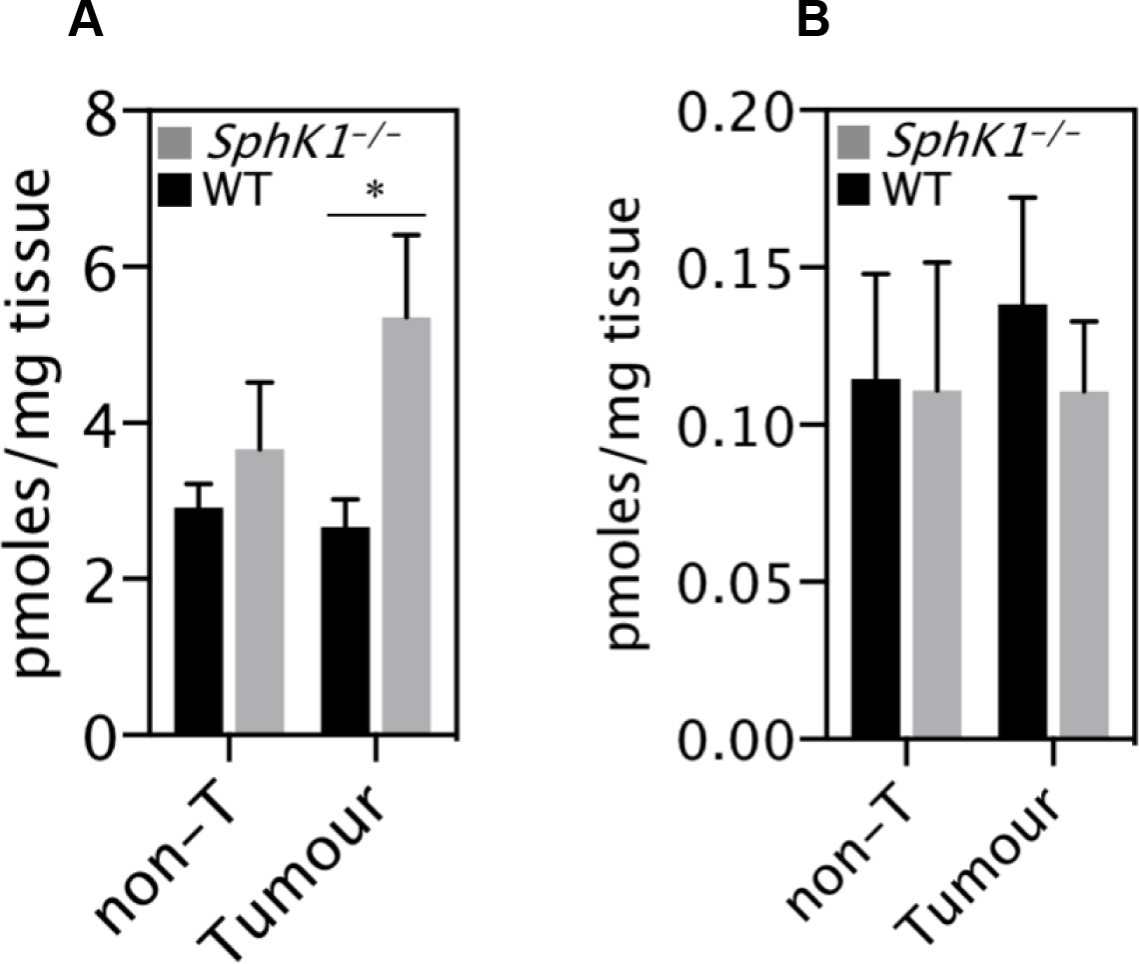
SphK1 deletion caused sphingosine accumulation in tumors (**A**) Sphingosine mass and (**B**) S1P were measured in tumor and non-tumor tissues of *Sphk1*^*−/−*^ and WT mice. Data are mean ± SEM (*n* = 6–7 per group). ^*^p < 0.05.

SphK1 converts sphingosine into S1P and both have been reported to play important roles in tumorigenesis [10, 11]. The level of sphingosine was significantly higher in tumors from SphK1^−/−^ mice than from WT mice (Figure 5A). However, the level of S1P was not significantly changed (Figure 5B), suggesting the inhibition of tumorigenesis caused by SphK1 deficiency is unlikely mediated by S1P.

### Deletion of SphK1 did not significantly alter the YAP or STAT3 signaling in tumors

**Figure 6:**
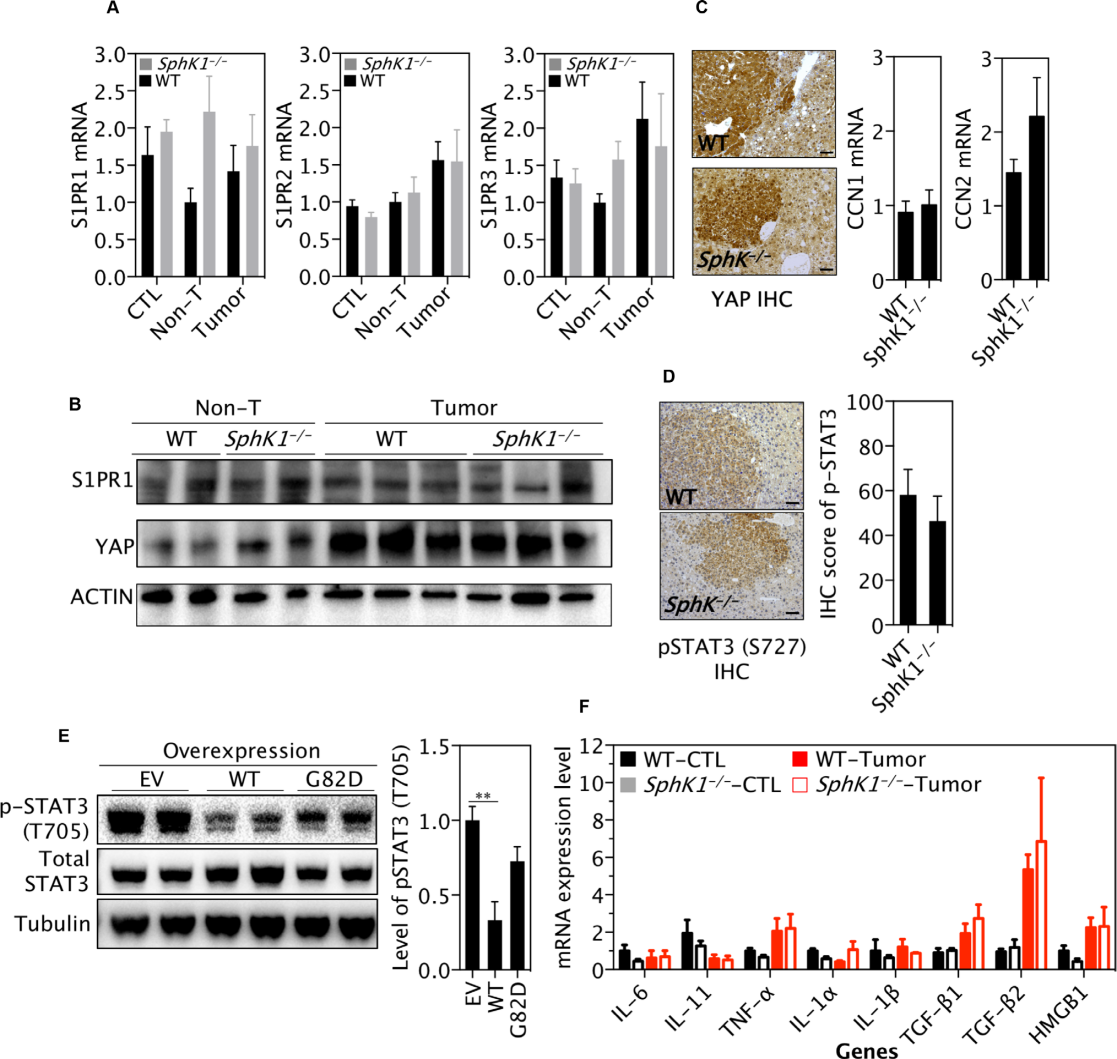
SphK1 deletion does not significantly alter the STAT3 or YAP pathway DEN was given once to WT and *SphK1*^*−/−*^ mice at postnatal day 14. Livers were harvested at 34 weeks post-DEN injection. (**A**) The mRNA Level of S1P receptor (S1PR). (**B**) Representative pictures of western blotting detecting YAP and S1PR1 (6 WT and 4 *SphK1*^*−/−*^ tumors). (**C**) YAP expression by immunohistochemistry (IHC) (left panel) and its targets by RT-qPCR (right 2 panels). Scale bars: 50μm. (**D**) Expression of phospho-STAT3 (T727) by IHC in liver tumors. Scale bars: 50μm. (**E**) Effect of SphK1 overexpression on STAT3 (2 of 4 biological repeats for each transfection) in HuH7 cells expressing empty vector (EV), wild type (WT) and dominant-negative (G82D) forms of SphK1. (**F**) Expression of cytokines by RT-qPCR. Normal livers (CTL, 8 WT and *SphK1*^*−/−*^) and tumor tissues (8 WT and 5 *SphK1*^*−/−*^) were used. non-T: non-tumor. Data represented as mean ± SEM, ^**^p < 0.01 (one-way ANOVA).

The crosstalk of IL-6/STAT3 and SphK1/S1P/S1PR pathways has been suggested to play an essential role in the progression of inflammation related tumors, such as colorectal cancer [10]. However, while we were able to confirm that levels of S1PR expression were up-regulated in tumors, there was no difference in mRNA levels of S1PR1, S1PR2 and S1PR3 between *SphK1*^*−/−*^ and WT tumors (Figure 6A and top panel in Figure 6B). Recently, S1P/S1PR2 has been shown to activate YAP [23, 24], a major oncogenic driver in liver cancer [25, 26]. Indeed, YAP expression was increased in liver tumors, but its level was not different between *SphK1*^*−/−*^ and WT tumors (Figure 6B, middle panel). Furthermore, SphK1 deletion has no effect on YAP activation, as reflected in its nuclear localization and expression of classic YAP target genes CCN1 and CCN2 (Figure 6C).

Immunohistochemical staining showed that the level of phospho-STAT3 (S727) was not significantly different between *SphK1*^*−/−*^ tumors and WT tumors (Figure 6D). In order to further test the effect of SphK1 on STAT3, vectors expressing wild type (WT) and mutant (G82D) SphK1 were transfected into Huh7 HCC cells and STAT3 protein was examined. Surprisingly, expression level of phospho-STAT3 (T705), which mainly mediates the oncogenic potential of STAT3 [27], was decreased by overexpressed SphK1 (Figure 6E). Indeed, as being a key downstream target gene, IL-6 expression was not significantly altered in *SphK1*^*−/−*^ tumors (Figure 6F). In addition, we did not find significant changes in mRNA levels of several key cytokines, including IL-11, IL-1α, IL-1β, TNF-α, TGF-β1, TGF-β2 and HMGB1 (Figure 6F). Considering these together with the unchanged expression level of S1PR1 (Figure 6A, 6B), the IL-6/STAT3/S1PR signaling pathway seems unlikely to play a major role in mediating the effect of SphK1 in DEN-induced liver carcinogenesis.

### Deletion of SphK1 reduced c-Myc expression in tumors

**Figure 7:**
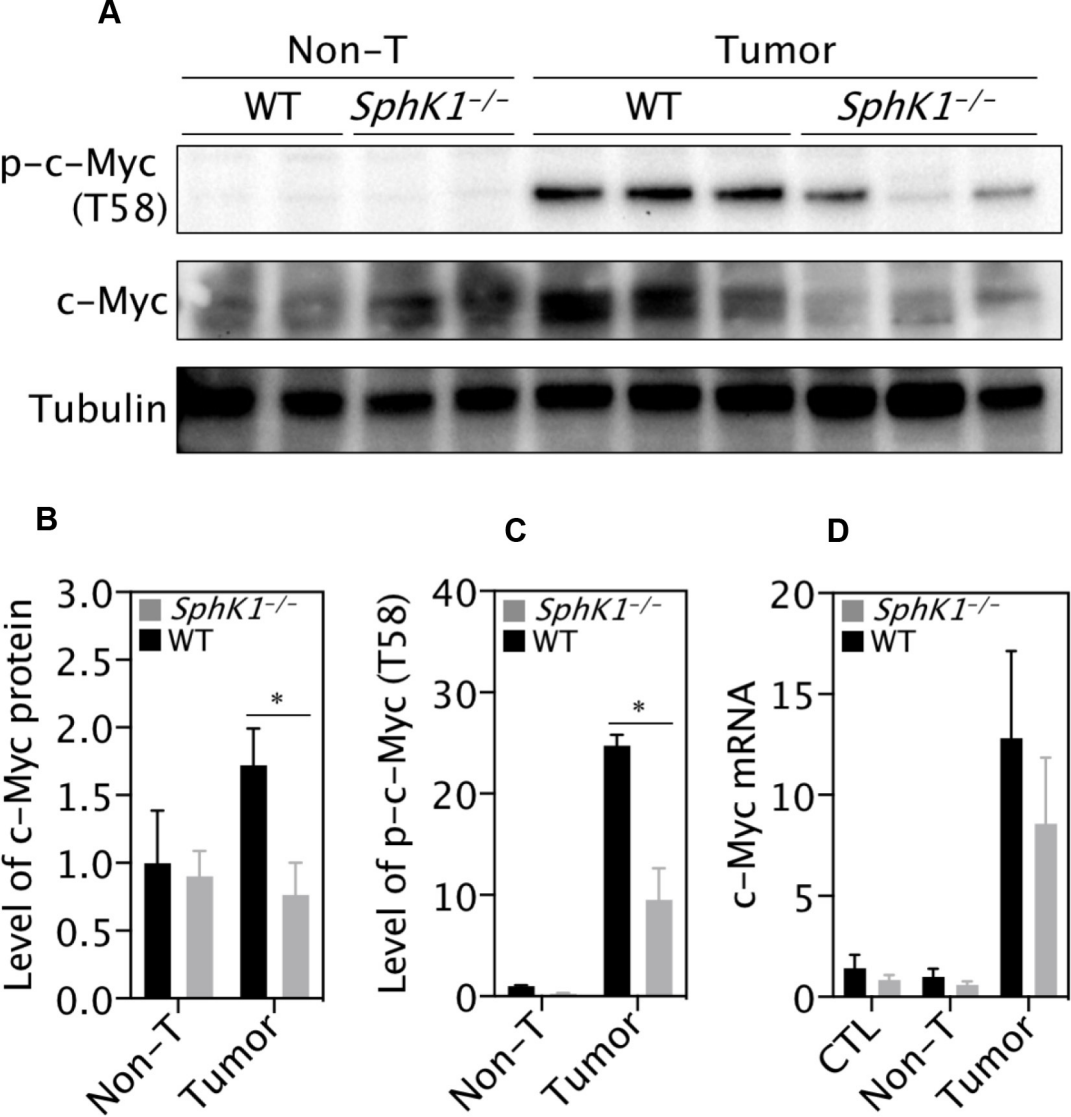
SphK1 deletion decreases level of c-Myc protein (**A**) Representative images of western blotting and (**B**, **C**) its quantification (normalization to Tubulin). There were 6 WT and 4 *SphK1*^*−/−*^ tumors. (**D**) Level of c-Myc mRNA detected by RT-qPCR. Normal livers (CTL, 8 WT and *SphK1*^*−/−*^), matched non-tumor (non-T) and tumor tissues (8 WT and 5 *SphK1*^*−/−*^) were used. Data represented as mean ± SEM, ^*^p < 0.05 (one-way ANOVA).

It has been well documented that c-Myc is a master driver of liver carcinogenesis [28]. The expression levels of both total and phospho-c-Myc (T58) were significantly up regulated in the DEN-induced liver tumors (Figure 7A–7C). Strikingly, SphK1 deletion significantly inhibited the expression of total and phospho-c-Myc (T58) (Figure 7A–7C). However, the level of c-Myc mRNA was not significantly different between WT and SphK1 tumors (Figure 7D), suggesting an effect of SphK1 on the post-transcriptional regulation of c-Myc.

### SphK1 expression is a prognostic factor for HCC patients’ survival

**Figure 8:**
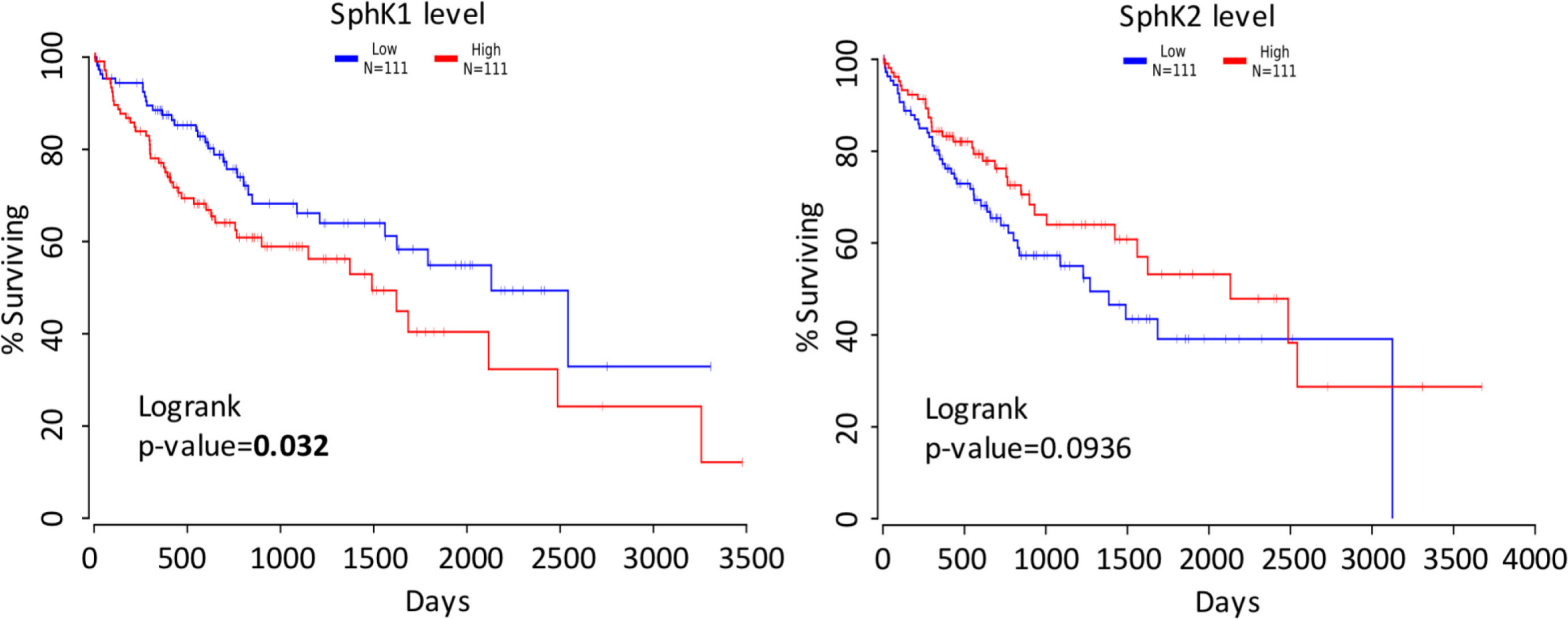
Survival analysis (**A**) Survival curves of HCC patients with high (red) and low (blue) SphK1 mRNA level. (**B**) Survival curves of HCC patients with high (red) and low (blue) SphK2 mRNA level. The raw data were obtained from the TCGA database and analyzed using the OncoLnc software.

Finally, we analyzed the TCGA study of human liver cancer and found that the survival of top one-third of HCC patients ranked by the SphK1 expression from the highest to lowest was statistically significantly worse than that of bottom one-third patient (Figure 8A). In contrast, SphK2 does not play a role in predicting the survival rate of HCC patients (Figure 8B).

## DISCUSSION

SphK1 deletion has been shown to inhibit the development of non-HCC tumors in p53 heterozygote mice [29], CRC [9, 10], and HNSCC in mice [30]. Thus, it might be intuitive that SphK1 would be involved in HCC pathogenesis. However, the molecular basis of HCC has been found to be heterogeneous and HCC occurs on a background of chronic liver injury. In addition, therapeutic approaches over the past decade just based on extrapolation of molecular targets from other cancers to HCC have largely failed. Hence it could not be automatically assumed that because SphK1 had been implicated these cancers that it would play a role in HCC development.

The novel pieces of data in the paper are as follows: (1) In a robust experimental HCC model the development and progression of HCC was significantly decreased in the *SphK1*^*−/−*^ mouse. (2) HCC in the *SphK1*^*−/−*^ mouse was unexpectedly associated with an increase in sphingosine rather than a decrease in S1P. (3) Downstream effects included a decrease in cellular proliferation and an increase in apoptosis and senescence. (4) Downstream pathways such as IL-6/STAT3 and YAP, usually associated with SphK1 related carcinogenesis, were not altered in the *SphK1*^*−/−*^ mouse HCC model. (5) C-myc was decreased at the protein but not mRNA level and finally (6) consistent with published data [31], lower levels of SphK1 (derived from the TGCA database) in human HCC were associated with improved survival. Thus, this study demonstrates that SphK1 is one of multiple known regulators of HCC development and progression.

Although we have not been able to precisely define the exact mechanism of the effect of SphK1 deletion on HCC development, several clues are provided from our studies that will become the basis of further work. We have firstly shown that SphK1 deficient tumor cells exhibited an increase in apoptosis and senescence and a decrease in proliferation. It has been widely reported that SphK1 promotes cancer growth via direct stimulation of proliferation and survival pathways [14]. Its deficiency not only inhibits the growth of cancer cells, but also enhances apoptosis. It has been previously reported that SphK1 deletion restored cellular senescence caused by p53 knockout thus inhibiting tumor growth [29]. Consistent with this, the present study showed deletion of SphK1 increased cellular senescence, as p16, a key player in mediating cellular senescence, was expressed higher in *SphK1*^*−/−*^ tumors than WT tumors. This scenario was confirmed in MEF cells. The SphK1 deletion-promoted senescence may function as a barrier against malignant transformation and prevent the expansion of precancerous cells.

The alteration of cell proliferation and death caused by SphK1 knockout in liver cancers could be attributed to changes of S1P, sphingosine or other sphingolipids. The function of SphK1 is to convert sphingosine into S1P, which helps to maintain the homeostasis of sphingolipid metabolism [14]. Consistent with a previous study [11], sphingosine was increased in SphK1 deleted tumors and S1P was not significantly altered. It is known that increased sphingosine levels induce cellular apoptosis [12, 32, 33]. Thus, it is likely that SphK1 deletion-caused inhibition of hepatocarcinogenesis was attributed to the accumulation of sphingosine or its upstream products but not S1P. This is in contrast to studies in other cancers where sphingosine-1-phosphate (S1P) diverse signalling through five S1P receptors (S1PR) or other intracellular targets has been implicated [5, 7, 13]. However, recent studies show that (i) up-regulated ceramides and sphingosine, not S1P, correlate significantly with alpha fetoprotein in serum of HCC patients and (ii) highly potent and selective SphK1 inhibitors may not affect cancer cell proliferation or survival [14, 34], thus supporting the concept that regulation through sphingosine itself might play a more important role in oncogenic transformation.

The role of c-Myc as a central regulator of hepatocarcinogenesis has been widely reported [28, 35]. Inhibition of c-Myc expression resulted in regression of established liver tumors, and hepatocyte-specific c-Myc-knockout mice showed much lower incidence of liver tumor compared to WT mice [36]. Consistent with these findings, the present study showed c-Myc protein expression was significantly increased in WT tumors, but was dramatically reduced in *SphK1*^*−/−*^ tumors. There was little or no change in c-Myc mRNA levels between WT and *SphK1*^*−/−*^ liver tumors, suggesting the inhibition of c-Myc expression is likely via post-transcriptional regulation mechanisms. Interestingly, it has been reported that SphK1 overexpression did not increase the level of c-Myc mRNA, but enhanced c-Myc mRNA translation and increased the growth of normal intestinal epithelial cells *in vitro* [37]. It has also been reported that c-Myc is directly or indirectly regulated by either sphingosine or ceramides [11, 37–39]. In addition, loss of c-Myc that resulted in the induction of p16 and variably p21 has been associated with cellular senescence [40, 41]. Indeed, we found massive senescence in cultured *SphK1*^*−/−*^ tumor cells as well as MEF. Thus, we would suggest that SphK1 deletion induced reduction of c-Myc and enhanced senescence contribute to the inhibition on liver tumorigenesis. Unfortunately, primary *SphK1*^*−/−*^ MEFs underwent rapid and large-scale senescence and cell death, so we did not have the opportunity to overexpress c-Myc as a means to rescue SphK1 deficiency-induced senescence. Further studies are needed to investigate whether and how SphK1 deletion inhibits c-Myc protein expression.

These results also need to be evaluated in the context of a series of negative mechanistic results in our work. Previous studies have reported that the IL-6/STAT3/S1PR signaling was a key cascade mediating the effect of overexpressed SphK1 on CRC tumorigenesis [10]. However, the present study shows the IL-6/STAT3/S1PR pathway appears to not be implicated in the inhibitory effect of SphK1 deficiency on liver carcinogenesis, but also many other signaling molecules such as TNF-α, IL-1α, IL-1β, TGF-β1 and HMGB1, which were reported in previous studies to play essential roles in the development of liver cancer [42–45], were not altered by SphK1 deletion. The reason for this difference may be attributed to different tumor models used in those studies in which the inflammatory agent DSS was used to promote CRC progression [10], and no similar promoters were employed in our DEN-induced liver cancer model. Furthermore, SphK1 might have organ specific effects on tumorigenesis.

In addition, we show that protein level of YAP, a key driver of liver carcinogenesis [25], was not changed by SphK1 deletion. It has been reported that YAP together with Notch, but independent of STAT3, mediates aberrant intestinal differentiation in gp130^Act^ mice [46]. Further STAT3 or YAP could up-regulate the transcription of c-Myc [47, 48]. However, neither of these pathways was altered with SphK1 deletion in DEN-induced liver tumors. Thus, this strongly argues for a STAT3 and YAP-independent pathway to mediate the effect of SphK1 on liver tumorigenesis in mice.

Taken together, this study has demonstrated that SphK1 deletion inhibits liver tumorigenesis in a robust mouse model of HCC that resembles aggressive human HCC [49–51]. The underlying mechanisms for this inhibition are not through the IL-6/STAT3/S1PR or YAP signaling pathways, but through other pathways among which the reduction of c-Myc protein level in SphK1 deficient tumors is especially interesting. This suggests that the downstream signaling pathways impacting on the development of HCC via SphK1 are organ specific providing further evidence that simply transferring known oncogenic molecular pathway targeting into HCC is not always valid. Further investigations are needed and warranted to thoroughly explore the underlying precise molecular mechanisms, with the help of cell-type-specific SphK1 knockout and knockin mice. The role of SphK1 in human HCC is supported by the correlation of low levels with increased patient survival [31, 52]. Interestingly, a compound called icaritin (PubChem CID: 5318980) has been reported to reduce HCC growth *in vitro* and *in vivo* by inhibiting SphK1 and ceramide accumulation [15]. Thus, SphK1 maybe a potential therapeutic target in primary liver cancer.

## MATERIALS AND METHODS

### Materials

Diethylnitrosamine (cat # N0258), crystal violet (cat # C0775), collagenase IV (cat # C5138), hydrocortisone (cat # H2270), insulin (cat # I9278), antibodies against actin (cat # A2103) and beta-tubulin (cat # T8328) were purchased from Sigma–Aldrich (Sydney, Australia). Antibodies against SphK1 (cat # ab71700 or ab61148), alpha-1-fetoprotein (AFP) (cat # ab46799), or phospho-T58 c-Myc (cat # ab28842) were bought from Abcam (Cambridge, CB4 0FL, UK). Antibody against c-Myc (cat # sc-788) and S1PR1 (cat # sc-25489) were purchased from Santa Cruz Biotechnology (Dallas, Texas 75220, USA). Antibodies against YAP (cat # 14074), PCNA (cat # 13110), STAT3 (cat # 9139), phospho-T705 STAT3 (cat # 9145) and phospho-T727 STAT3 (cat # 9134) were obtained from Cell Signaling Technology (Danvers, MA, USA).

### Animals and DEN-induced liver cancer mouse model

Animal experiment protocols were approved by the University of Sydney Animal Ethics Committee and Sydney Local Hospital District Animal Welfare Committee, and were in accordance with the NSW Animal Research Act 1985. Mice were housed in a temperature-controlled pathogen-free environment on a 12-hour light and 12-hour dark cycle, had *ad libitum* access to food and water. SphK1 knockout (*SphK1*^−/−^) and litter mate wild type (*WT*) mice that were derived and backcrossed on a C57BL/6N background were used.

DEN-induced primary liver cancer mouse model was made according to a previously established protocol [16, 20, 53]. Briefly, DEN (25 mg/kg, once) was injected intraperitoneally (i.p.) into 14-day-old male pups. Mice were euthanized by CO_2_ inhalation at 19 and 34 weeks after DEN treatment. Visible tumors (≥0.5 mm) on the surface of each liver were counted and measured with a caliper. The tumor, its surrounding non-tumor tissues and age-matched healthy liver tissues were collected and either fixed in 10% neutral buffered formalin or snap frozen for further analysis.

### Measurement of sphingosine and S1P

Sphingolipids were extracted from murine liver tumour and non-tumour tissues and then quantified with HPLC-MS/MS as reported before [17]. Briefly, tissues were homogenized in lipid extraction buffer (isopropanol/water/ethyl acetate at 30:10:60, v/v) and dried in a SpeedVac system (Thermo, Waltham, MA, USA) after adding internal standards (C17-sphingosine and C17-S1P). The dry lipid extracts were reconstituted in the HPLC mobile phase, i.e. 1 mM ammonium formate and 0.2% (v/v) formic acid in a methanol and deionized water mixture (80:20, v/v). Sphingosine and S1P were then quantified using HPLC-MS/MS.

### Histological and immunohistochemical staining

Tissue sections (4μm) from paraffin embedded livers were cut and stained with haematoxylin and eosin (H&E) as described previously [54, 55]. Tumors were identified on H&E sections and quantified as described previously [56]. In brief, the number of foci in each liver section and the area (mm^2^) of each liver section was determined and expressed as the number of foci per mm^2^.

The expression of SphK1, AFP, PCNA, YAP and STAT3 protein was analyzed with immunohistochemistry. TUNEL (Roche) and immunohistochemical stainings were performed as described before [57]. TUNEL- or PCNA-positive cells were quantified with the help of FIJI software and expressed as the number of positive cells per mm^2^ or percentage of total cells, but semi-quantitative immunohistochemical assay was applied to phospho-STAT3 (T727) as reported before [58]. Briefly, the intensity of staining was graded as negative (0), weak (1), moderate (2) and strong (3), the percentage of positive cells were measured, then the IHC score is calculated by the formula: 3 × percentage of strong staining + 2 × percentage of moderate staining + percentage of weak staining. Only the intensity of staining was applied to SphK1 as which shows even staining.

### RNA isolation and reverse-transcription polymerase chain reaction (RT-PCR)

Total RNA was extracted from liver tissues or cells with TRIzol (Life technologies, NY, USA). The complementary DNA (cDNA) was generated from total RNA with high capacity reverse transcription kits (Life Technologies) and also no template controls were made by omitting reverse transcriptase. The abundance of transcripts was assessed by quantitative real-time PCR (qPCR) on a Corbett Rotor-Gene 3000 with SsoFast EvaGreen (Bio-Rad, Hercules, CA, USA). The level of each transcript was determined by a standard curve. The expression data for each of interested genes was normalized for the efficiency of amplification with a set of reference genes, i.e. cyclophilin A, HPRT and RPL13A. PCR primers were synthesized by Sigma–Aldrich and listed in the supplementary data (Supplementary Table 1).

### Protein extraction and western blotting

Protein extraction and Western blotting was performed as described previously [59, 60], but the Gel Doc XR+ System from Bio-Rad (Hercules, California 94547, USA) was used to capture digital images, instead of photographic films.

### Primary mouse embryo fibroblast (MEF) and liver tumor cells

SphK1^−/−^ and WT mouse embryos at E14.5 were harvested and then the isolation and culturing protocols for mouse embryonic fibroblasts (MEFs) described before by Xu were adapted [21, 22]. In brief, 7.5 × 10^5^ MEF cells per passage were plated in a 25cm^2^ culture flask, incubated for 3 days or longer depending on their growth and then were passaged again instead of standard 3T3 protocol, i.e. MEF were passaged every 3 days and inoculated at 3 × 10^5^ cells per 20 cm^2^ of culture dishes continuously. Primary tumor cells were isolated from liver tumors dissected out from mice at 34 weeks post-DEN injection and cultured according to protocols as reported previously [61]. Briefly, tumors were minced, washed with ice-cold PBS, and then incubated with Collagenase IV (0.05% in Hank’s BSS with Ca^2+^, pH = 7.4, C5138, Sigma–Aldrich) digestion buffer at 37°C with gentle stirring for up to 1 hour. Cells were pelleted by centrifugation at 50 g for 4 minutes after being filtered through a 70 μm cell strainer. After cell pellets were washed once with PBS, cells were re-suspended with growth medium (DMEM/F12 with 20% heat inactivated fetal bovine serum, 1% L-glutamine, 1% penicillin-streptomycin, 0.01 g/L hydrocortisone hemisuccinate, 0.01 g/L insulin and 20 μg/L EGF) and plated in several 25 cm^2^ culture flasks (termed as passage 0). Upon confluent, cells would be trypsinized and passaged for experiments or frozen for usage in future. All of cells were cultured at 37° C with 5% CO_2_ and 95% air.

### The soft agar colony formation assay

Malignancy of tumor cells was detected with the soft agar colony formation assay as reported previously [6]. Briefly, single-cell suspensions of *SphK1*^*−/−*^ and WT tumor cells were plated into 24-well plates (2,000 cells per well) in 400 μL of growth media with 0.33% agar on a layer of 400 μL of the same medium containing 0.5% agar. At 2 weeks post-plating, cells were stained with 0.005% crystal violet-2% ethanol in PBS and the colonies (more than 8 cells) were counted under a microscope.

### Measurement of senescence-associated ß-galactosidase (SA-ß-gal) activity

The Senescence β-Galactosidase Staining Kit (cat # 9860, Cell Signaling Technology, USA) was used to detect cells with active SA-ß-gal. Cells with blue-colored staining for SA-β-gal activity were counted under a bright field microscope and expressed as the percentage of total cells.

### Survival analysis

HCC patients in the TCGA database (https://peerj.com/articles/cs-67/) were ranked by the level of SphK1 expression (highest to lowest) and divided into two groups, i.e. top one-third and low one-third. These groups were analyzed by using OncoLnc (http://www.oncolnc.org) [62].

### Statistical analysis

All data are expressed as mean ± SEM. After data transformation if needed, analysis of variance (ANOVA) was used to analyze the differences among groups, *p* < 0.05 was regarded statistically significant.

## SUPPLEMENTARY MATERIALS TABLE



## Abbreviations

SphK: sphingosine kinase
