# Supplementary material for: Deletion of sphingosine kinase 1 inhibits liver tumorigenesis in diethylnitrosamine-treated mice
Source: Oncotarget. 2018 Feb 26;9(21):15635–49. doi: 10.18632/oncotarget.24583 (PMC5884653; doi:10.18632/oncotarget.24583)
Supplement: Supplementary file 1 [file oncotarget-09-15635-s001.pdf]

Deletion of sphingosine kinase 1 inhibits liver tumorigenesis in diethylnitrosamine-treated mice

SUPPLEMENTARY MATERIALS

Supplementary Table: 1 Sequences of primers used for real-time PCR

Gene	Forward primer	Reverse primer
SGPP1	GCCATGTTCCTGCTCACCTA	AGTGAACTCCAGCAGGGAATG
SGPP2	GGAGCCTGCGGGATTCACA	AGGGTCGATATTCCAGTGGGT
SGPL1	ATGGGGGACCAGATTCCTGT	TAACGCCAAGTCCCGGTAAG
SphK1	ATGCATGAGGTGGTGAATGG	CCCAGCATAGTGGTTCACAG
SphK2	CACTTATGAGGAGAATCGTGCA	TCTGTCGTTCTGTCTGTATGAG
Afp	ATTCACGCAGAGAATGGCGT	CTGAGACAGGAAGGTTGGGG
p15	AGATCCCAACGCCCTGAAC	CAGTTGGGTTCTGCTCCGT
p16	AAAGCGAACTCGAGGAGAGC	CGTGAACGTTGCCCATCATC
p19	CGTGAACATGTTGTTGAGGC	TCGAATCTGCACCGTAGTTG
p21	CGGTGTCAGAGTCTAGGGGA	ATCACCAGGATTGGACATGG
p53	CTCCCCCGCAAAAGAAAA	TATGGCGGGAAGTAGACTGG
ccnd1	AAAATGCCAGAGGCGGATGA	GAAAGTGCGTTGTGCGGTAG
S1PR1	GCATTGGATTGCTCGCTGAC	GGGGTGGTATTTCTCCAGGC
S1PR2	AAAACCAACCACTGGCTGTC	CTCTGAGTATAAGCCGCCCA
S1PR3	TCTTAGCTGAGACACGGCAG	ACAATTGGGAGAGTGGTGGT
IL-6	GGGAAATCGTGGAAATGAGA	TTCTGCAAGTGCATCATCGT
IL-11	TTGATGTCCTACCTCCGGCAT	CAGGGGGATCACAGGTTGGT
TNF-α	CCACCACGCTCTTCTGTCTA	CACTTGGTGGTTTGCTACGA
IL-1α	ACGTCAAGCAACGGGAAGAT	AAGGTGCTGATCTGGGTTGG
IL-1β	AGACAACTGCACTACAGGCTC	TTGTCGTTGCTTGGTTCTCCT
TGF-β1	ACTGGAGTTGTACGGCAGTG	GGGGCTGATCCCGTTGATTT
TGF-β2	CCCGGAGGTGATTTCCATCT	TGTAGAAAGTGGGCGGGATG
HMGB1	TACAGAGCGGAGAGAGTGAG	TGCCTCTCGGCTTTTTAGGA
CCN1	CTTCCTGTCTTTGGCACCGA	CTCGTGTGGAGATGCCAGTT
CCN2	CCTAGCTGCCTACCGACTG	TTAGAACAGGCGCTCCACTC
c-Myc	TCCTGTACCTCGTCCGATTC	GGTTTGCCTCTTCTCCACAG
cyclophilin A	CAAATGCTGGACCAAACACAAACG	GTTCATGCCTTCTTTCACCTTCCC
HPRT	AGCTACTGTAATGATCAGTCAACG	AGAGGTCCTTTTCACCAGCA
RPL13A	CACTCTGGAGGAGAAACGGAAGG	GCAGGCATGAGGCAAACAGTC
